# Supplementary material for: Small Molecule Cyclotriazadisulfonamide Abrogates the Upregulation of the Human Receptors CD4 and 4-1BB and Suppresses In Vitro Activation and Proliferation of T Lymphocytes
Source: Front Immunol. 2021 Apr 21;12:650731. doi: 10.3389/fimmu.2021.650731 (PMC8097030; doi:10.3389/fimmu.2021.650731)
Supplement: Supplementary file 1 [file DataSheet_1.pdf]

SUPPLEMENTAL FIGURES

Supplementary Figure 1. CADA has no effect on superantigen-activated Jurkat cells and does not exert cytotoxicity (Related to Figure 1).

(A) Jurkat cells were pre-treated with CADA (10 µM) for 2 days and subsequently activated with Raji-GFP cells loaded with staphylococcal enterotoxin E (SEE) or staphylococcal enterotoxin B (SEB). Cell surface CD69 and CD4 expression was measured 24h later. Nonactivated cells are represented in blue, activated cells with DMSO in black, activated cells with CADA in red, and unstained control in grey.

(B) Same as in (A). Bar diagrams are showing the CD69 expression (MFI) of unstimulated and SEE-stimulated cells on day 1 post activation, with DMSO in white and CADA in red. Values represent mean ± SD of five independent experiments (n=5).

(C) Jurkat cells were incubated with different concentrations of CADA or MMF during 2 days. Next, cells were stained with trypan blue and counted with a Vi-CELL device (top panel), or MTS-PES was added to measure cellular metabolic activity, and read-out was done 2h later on a spectrophotometer (bottom panel). Cell viability and metabolic activity of cells is given as percentage of untreated control. Multiple t-tests were performed to compare each concentration of CADA or MMF to the corresponding DMSO control with $^*p<0.05$ and with Holm-Sidak method as correction for multiple comparison. Curves are showing mean values ± SD of four (top panel; n=4) or ten repeat experiments (bottom panel; n=10).

Supplementary Figure 2. Reduced CD4 surface expression affects lymphocyte proliferation in the MLR (Related to Figure 1).

PBMCs were co-cultured with mitomycin C inactivated RPMI1788 cells in the presence of CADA (left panel) or the anti-CD4 antibody Clenoliximab (right panel). At day 5, one sample was used to determine cell surface human CD4 expression using flow cytometry. In parallel, [^{3}H]-thymidine was added to an identical sample and proliferation response was measured by detecting counts per minute 18h later. To avoid steric hindrance for the detection of CD4, the monoclonal anti-human CD4 antibody clone OKT4 was used as this antibody binds to the D3 domain of CD4, while Clenoliximab binds to the D1 domain. Human CD4 expression (blue open symbols with dotted line), given as percentage of untreated control, is plotted on the left Y-axis, and lymphocyte proliferation (red solid symbols with solid line), given as percentage of DMSO control for CADA and as percentage of ProClin 300 control for Clenoliximab is plotted on the right Y-axis. Curves are showing mean values ± SD of four different donors (n=4).

Supplementary Figure 3. CADA reduces basal cell surface expression level of CD4 and CD8 but not that of CD3 and TCR, and suppresses receptor upregulation after stimulation by CD3/CD28 beads or PHA (Related to Figure 2).

(A) PBMCs were treated and collected as described in the legend to Figure 2B. CD4 (blue) and CD8 (green) receptor expression analysis was performed on the samples from Fig. 2B and 2C, respectively, for the time points 0h (d0), day 1 (d1) and day 4 (d4) post activation. For each donor, the MFI value of receptor expression (acquired from at least 5,000 cells per donor) in the CADA-treated sample is normalized to the matching DMSO control of that specific time point to calculate the relative receptor expression at each time point post stimulation. A paired t-test was performed to compare CADA to DMSO with *$p<0.05$. Bars are mean ± SD; n=4.

(B) PBMCs (from the same donors of Figure 2D) were stimulated with PHA for 3 days and were subsequently exposed to CADA (10 µM) or DMSO for another 2 days. Cells were analyzed for cell surface receptors CD3, CD4, CD8 and TCRα/β. Bar diagrams are showing the MFI of the indicated receptor on gated $CD4^+$ or $CD8^+$ subpopulations, with DMSO in white and CADA in red. Welch's corrected t-tests were performed to compare CADA to DMSO with *$p<0.05$. Values represent mean ± SD of two different donors (n=2).

(C) PBMCs were pre-incubated with CADA (10 µM) or DMSO for 3 days, after which they were activated by CD3/CD28 beads or PHA. At day 2 post activation, cells were analyzed for cell surface receptors CD3 and TCRα/β. Bar diagrams are showing the MFI of the indicated receptor, with DMSO in white and CADA in red. Welch's corrected t-tests were performed to compare CADA to DMSO with *$p<0.05$. Values represent mean ± SD of four different donors (n=4).

(D) Same as in (C) but for the analysis of CD69 expression on day 1 post activation. Representative dot plots show CD69/CD4 double staining for unstimulated and CD3/CD28 beads-stimulated cells.

(E) Same as in (C) but for the analysis of CD69 expression at 4h and 24h (1d) post activation. Bar diagrams are showing the MFI of CD69 receptor expression, with DMSO in white and CADA in red. Welch's corrected t-tests were performed to compare CADA to DMSO with *$p<0.05$. Values represent mean ± SD of four to eight different donors (n=4 for the samples of 4h; n=6 for PHA at d1; n=8 for CD3/CD28 at d1).

Supplementary Figure 4. CADA differentially affects cytokine release by activated PBMCs in the MLR (Related to Figure 4).

PBMCs were stimulated with mitomycin C inactivated RPMI1788 cells exposed to CADA (10 µM). Supernatants were collected on day 5 post stimulation and cytokine levels were determined by Bio-Plex assay. Bar diagrams are showing the cytokine concentration (mean ± SD), with DMSO in white and CADA in red, and with individual values shown as open (DMSO)

or solid (CADA) symbols. Welch's corrected t-tests were performed to compare CADA to DMSO with *p<0.05. Samples were taken from four different donors (n=4).

Supplementary Figure 5. CADA treatment results in decreased CD25 upregulation and reduced intracellular pSTAT5 and CTPS1 levels in activated PBMCs. (Related to Figure 5).

(A) PBMCs were pre-incubated with CADA (10 µM) or DMSO for 3 days, after which they were activated with PHA or CD3/CD28 beads. Cellular surface CD25 expression was measured on gated $CD4^+$ and $CD8^+$ T cells by flow cytometry at 4h, 1 day, 2 days, 3 days or 4 days post activation. Graphs show individual MFI values of CD25 expression (acquired from at least 5,000 cells) from different donors with DMSO-treated cells as open symbols and CADA-treated cells as solid red dots. A paired t-test was performed to compare CADA to DMSO with *p<0.05. Horizontal lines indicate the mean values of the same donors from Figure 5A (n=4).

(B) CD25 receptor expression analysis was performed on the samples from (A) and from Fig. 5A for the time points 0h (d0), day 1 (d1) and day 4 (d4) post activation. For each donor, the MFI value of CD25 expression (acquired from at least 5,000 cells per donor) in the CADA-treated sample is normalized to the matching DMSO control of that specific time point to calculate the relative receptor expression at each time point post stimulation. Bars are mean ± SD; n=4.

(C) Representative dot plot of a sample from (A) showing CD25/CD4 double staining at 4h post CD3/CD28 beads-activation, with DMSO as black dots and with CADA as red overlay. Do note that the CADA-treated cells have similar CD25 expression levels as DMSO, but reduced CD4 levels (see difference in black and red population in upper quadrants).

(D) PBMCs were pre-incubated with 10 µM of CADA or DMSO for 3 days, after which they were activated by CD3/CD28 beads or PHA as indicated. Supernatant was taken 2, 3, or 4 days post activation and sCD25 levels were measured by ELISA. Data are shown for 4 donors of PBMCs (indicated separately) with DMSO-treated samples as open symbols and CADA-treated samples as solid red symbols. A paired t-test was performed to compare CADA to DMSO with *p<0.05. Horizontal lines indicate the mean values of four different donors (n=4).

(E) Representative dot plots of CD25/pSTAT5 double staining on a sample from Figure 5C.

Supplementary Figure 6. CADA reduces the upregulation of CD28 on activated lymphocytes (Related to Figure 6).

PBMCs were pre-incubated with 10 µM of CADA or DMSO for 3 days, after which they were activated by CD3/CD28 beads or PHA.

(**A**) Representative dot plots of CD28/CD4 double staining on beads-activated cells at 4h and 2d post stimulation.

(B) Cell surface CD28 expression was measured on gated $CD4^+$ (top panels) and $CD8^+$ (bottom panels) T cells by flow cytometry at 4h, 1 day, 2 days, 3 days or 4 days post activation. Graph shows individual MFI values of CD28 expression (acquired from at least 5,000 cells per donor) from different donors with DMSO-treated cells as open symbols and CADA-treated cells as solid red dots. A paired t-test was performed to compare CADA to DMSO with $*p<0.05$. Horizontal lines indicate the mean values of four different donors (n=4).

(**C**) Same as in (B) but for each donor, the MFI value of the CADA-treated sample is normalized to the corresponding DMSO control of that specific time point (set as 1.00) to calculate the relative CD28 receptor expression. Multiple t-tests were performed to compare CADA to DMSO for each condition with $*p<0.05$ and with Holm-Sidak method as correction for multiple comparison. Bars are mean ± SD; n=4.

Supplementary Figure 7. CADA differentially affects the protein expression levels of co-stimulatory receptors in transfected cells (Related to Figure 7).

(**A and B**) HEK293T cells were transiently transfected with different receptor constructs (as shown in Figure 7A) and incubated with CADA.

(A) Cells were transfected with hCD4tGFP-2A-RFP and given DMSO (CTR) or treated with CADA for 72h. In parallel, CADA-treatment was terminated after 24h (CADA wash). These cells were washed profoundly and given control medium for the duration of the experiment. At the indicated time points, cells were collected and tGFP (representing human CD4 levels) was measured by flow cytometry. The average MFI of tGFP is shown (mean ± SD; n=2). Of note is that the SD of the control samples (black curve) and CADA samples (green curve) is too small to be visible on the graph.

(B) Cellular expression of each receptor-tGFP-2A-RFP was determined by measuring tGFP levels by flow cytometry. Receptor levels in CADA-treated samples are normalized to the corresponding DMSO control. Values are mean ± SD. For each construct, a four parameter dose-response curve for CADA was fitted to data from at least three replicate experiments (n ≥ 3).

Supplementary Figure 8. Cell free *in vitro* translation/translocation assay to study the co-translational translocation of proteins (Related to Figure 8).

(A) Schematic representation of translating ribosomes targeting to the ER membrane. When the signal peptide (SP) is emerging from the translating ribosome (1), the ribosome-nascent chain complex is targeted to the ER membrane and will dock onto the Sec61 translocon. The SP is then inserted in the translocon and makes a looped structure with its N-terminus facing the cytosol (2). The signal peptidase, located at the lumenal side of the ER membrane, will cleave the SP from the pre-protein (3). Next, the oligosaccharyltransferase (OST), also located

at the lumenal side of the ER membrane, will add glycans to the protein (4). Finally, a lateral gate in the translocon facilitates the integration of hydrophobic transmembrane protein segments into the lipid bilayer for the accommodation of integral membrane proteins (5).

(B) Schematic overview of the protocol used in the translation/translocation assay.

Supplementary Figure 1

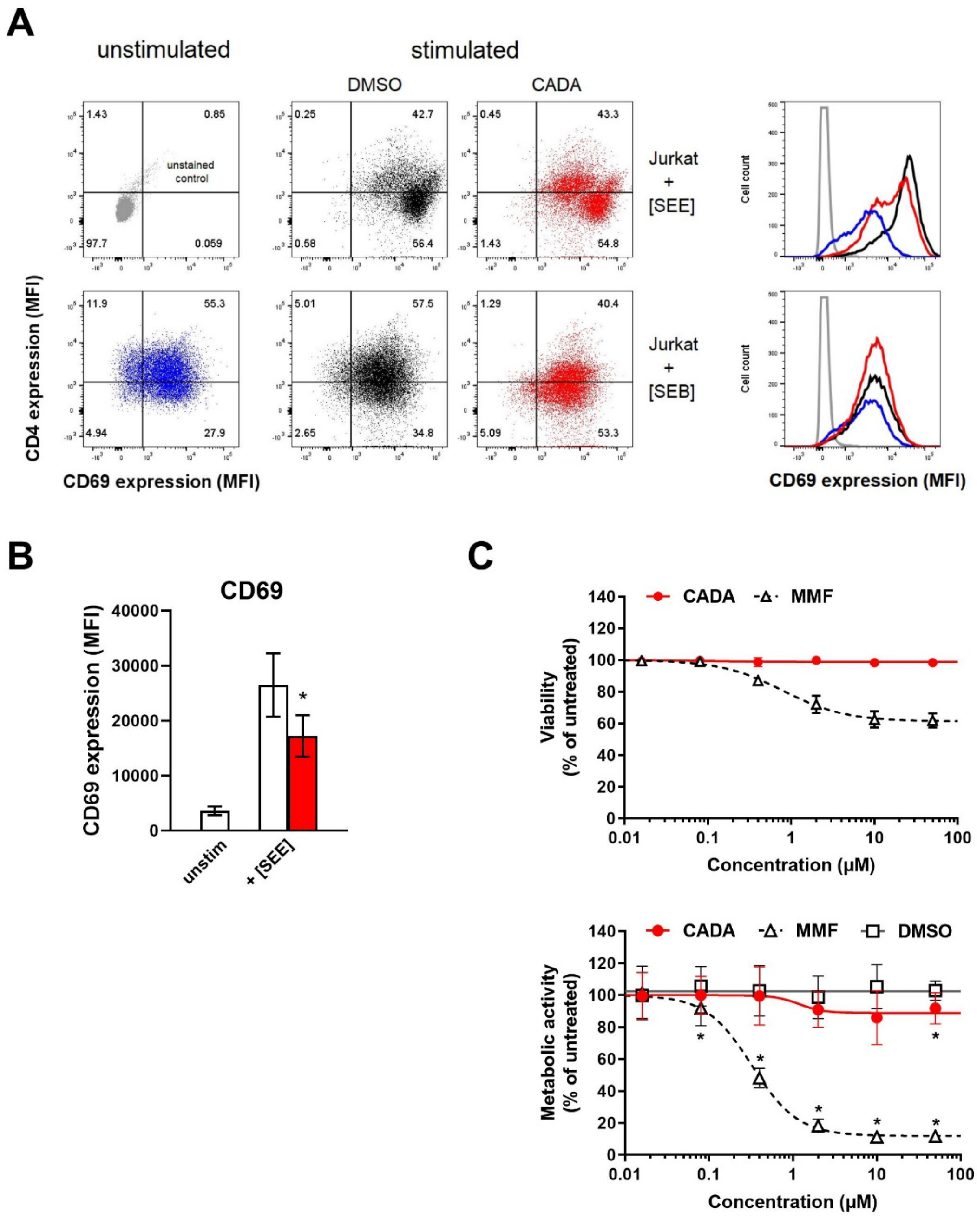

Claeys et al.

Supplementary Figure 2

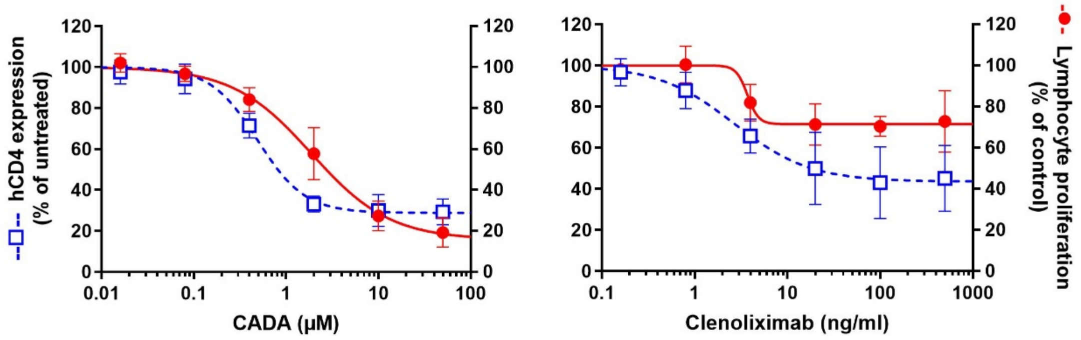

Claeys et al.

Supplementary Figure 3

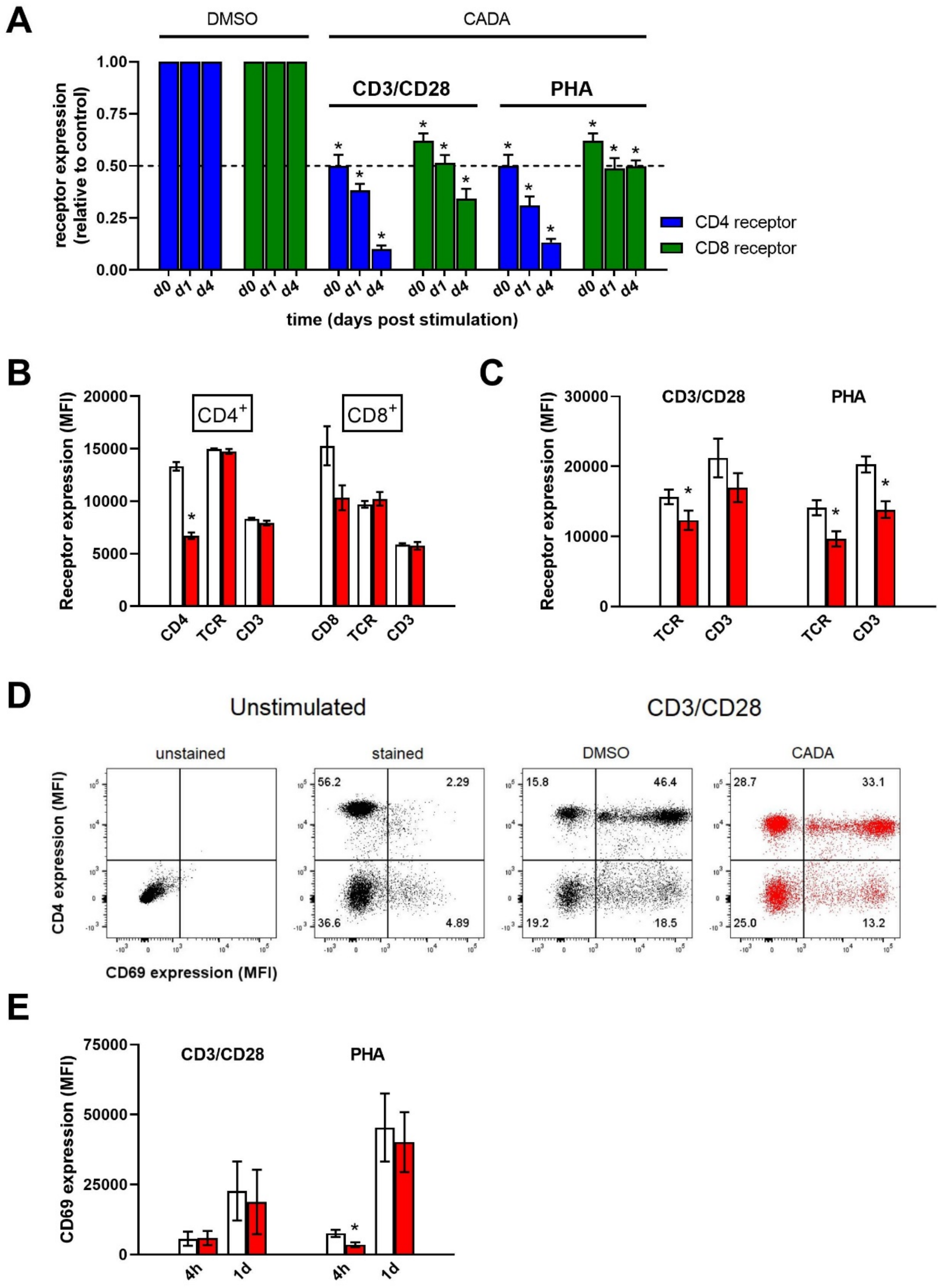

Claeys et al.

Supplementary Figure 4

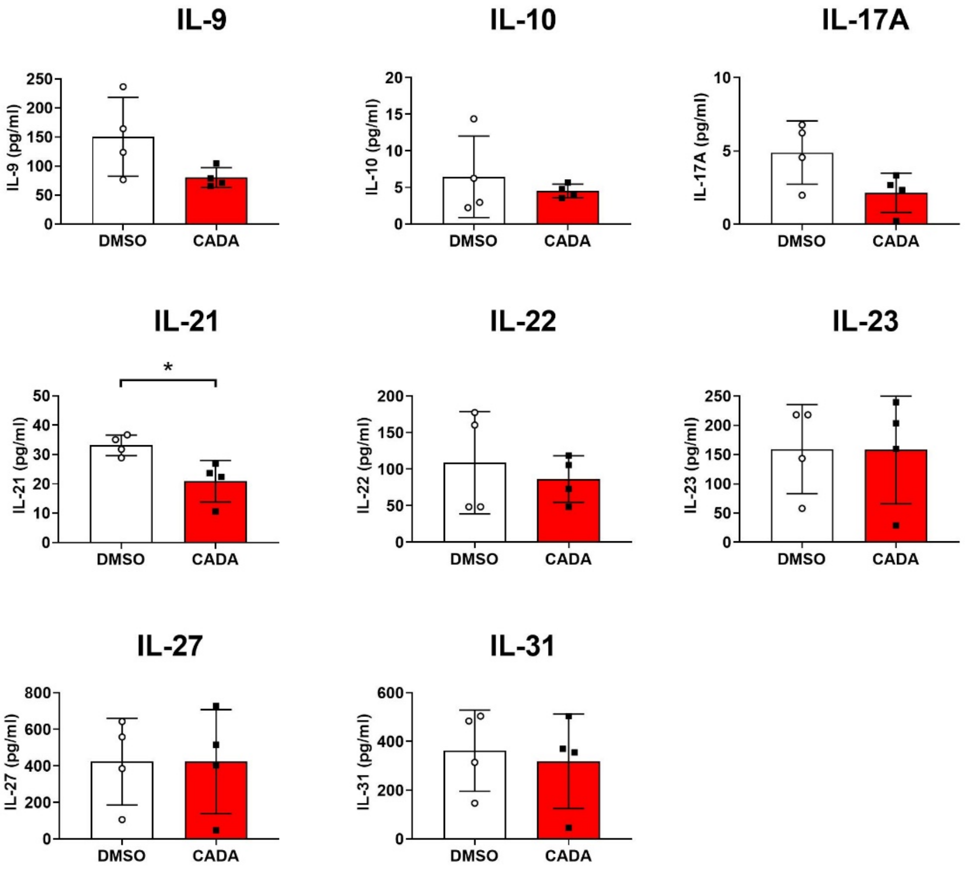

Claeys et al.

Supplementary Figure 5

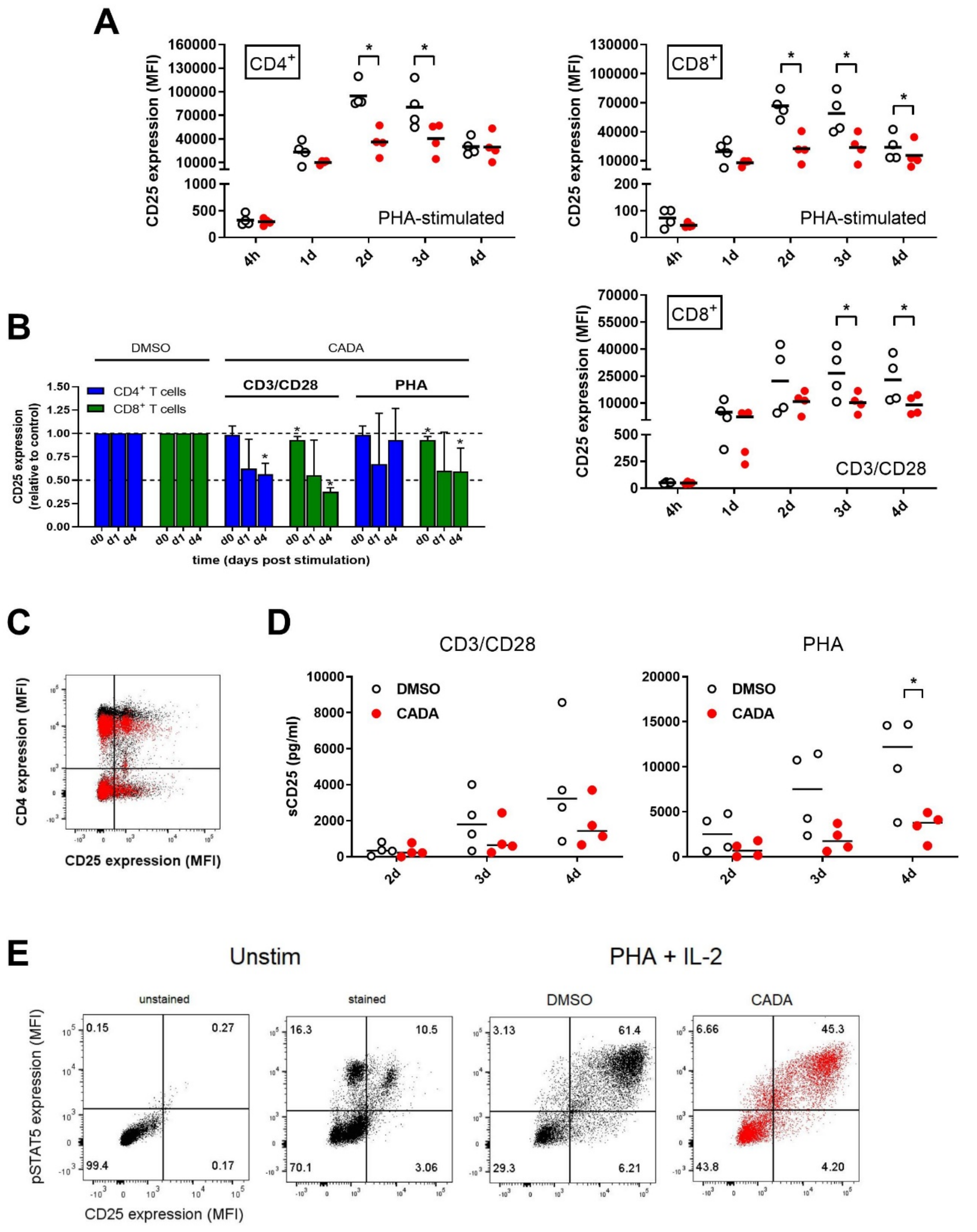

Claeys et al.

Supplementary Figure 6

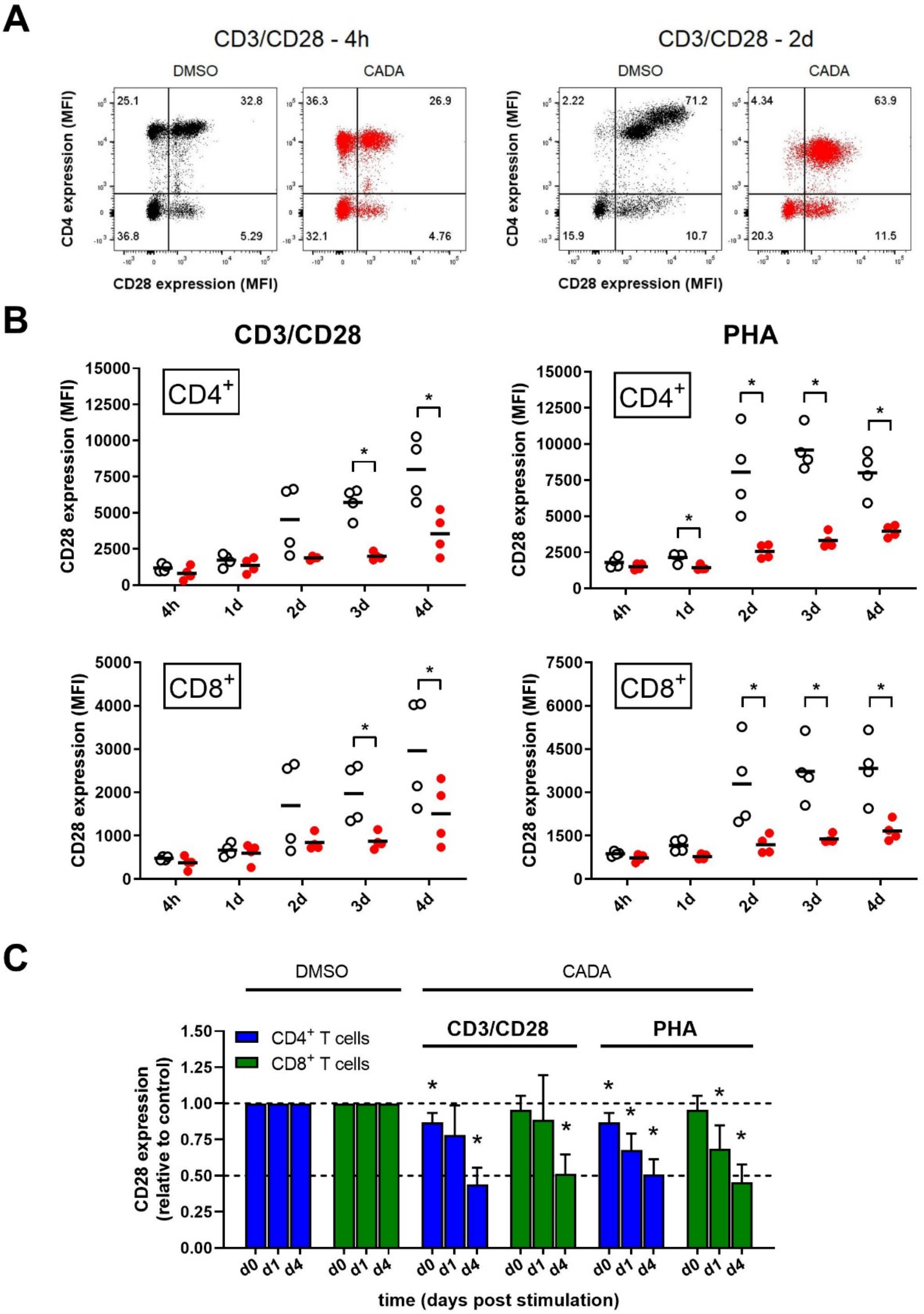

Claeys et al.

Supplementary Figure 7

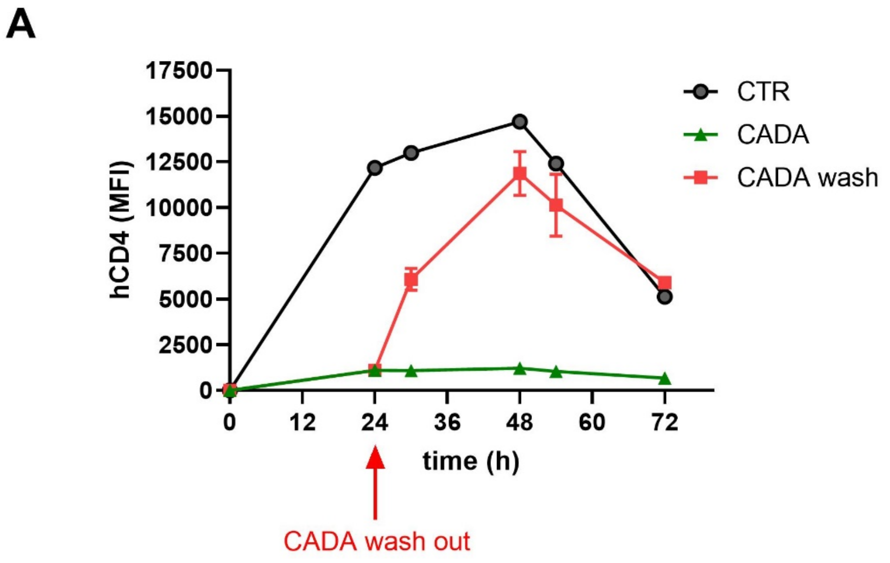

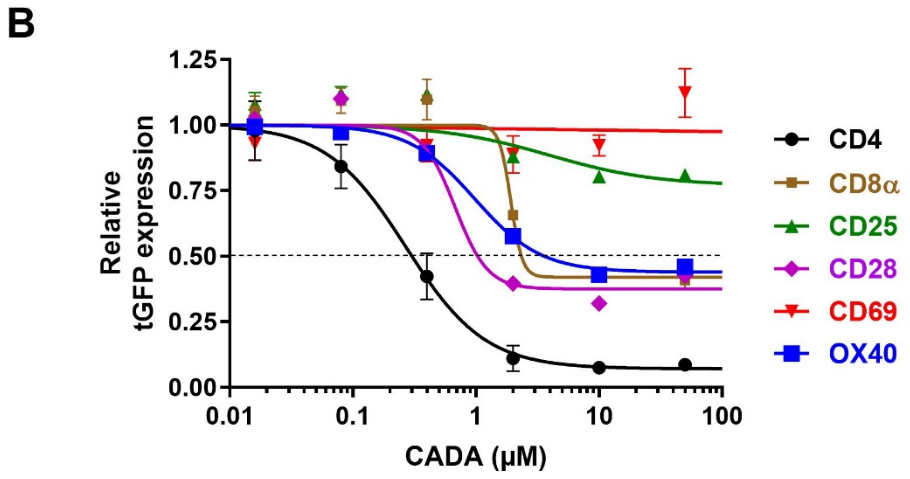

Claeys et al.

Supplementary Figure 8

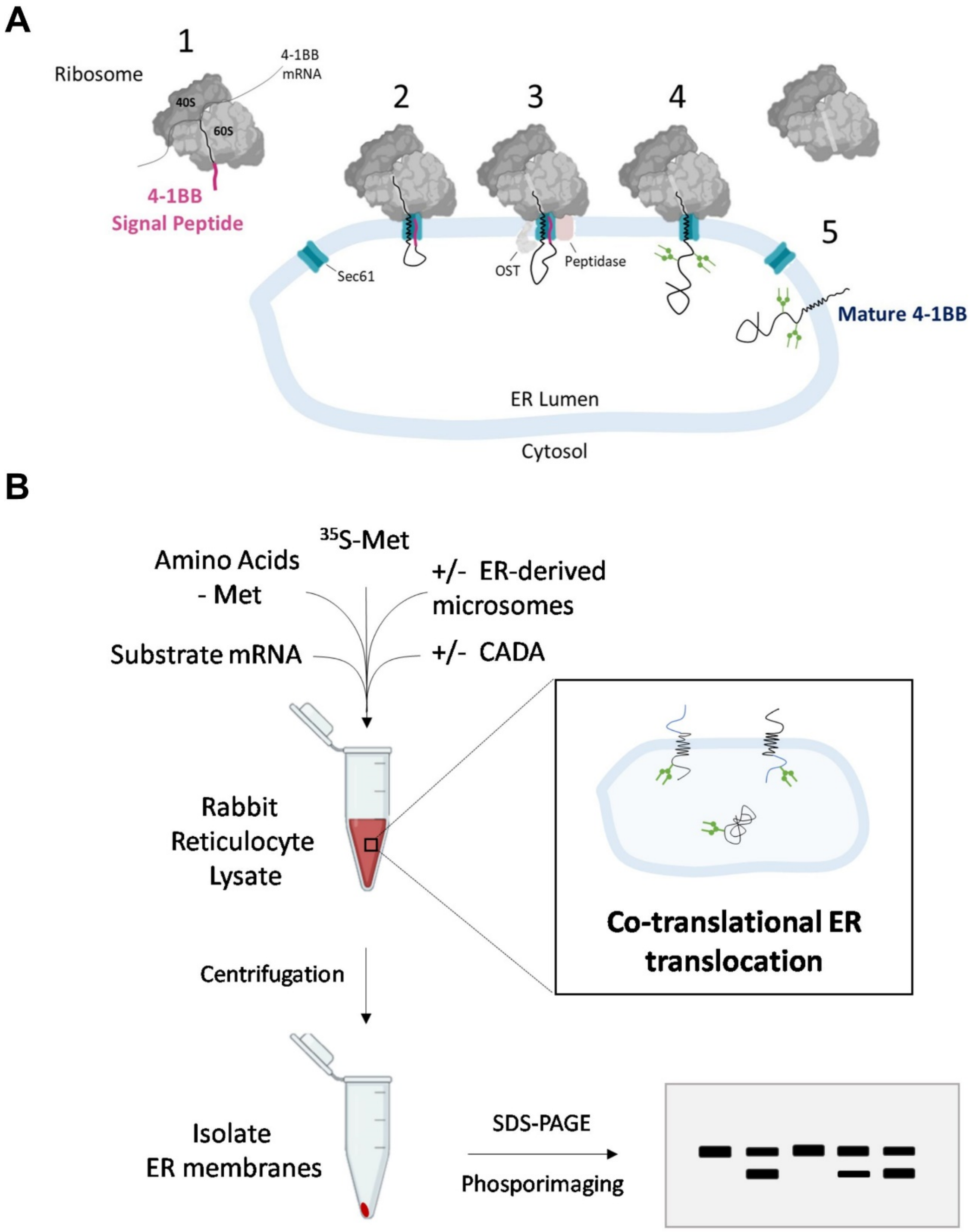

Claeys et al.
